# Adenotonsillectomy in facial growing patients: spontaneous dental effects

**DOI:** 10.1590/S1808-86942011000500011

**Published:** 2015-10-22

**Authors:** Silvia Regina Amorim Pereira, Silvia Fuerte Bakor, Luc Louis Maurice Weckx

**Affiliations:** 1 PhD in Sciences - UNIFESP, Coordinator of the Graduate Program in Orthodontics of the School of Pindamonhangaba. Professor - Department of Graduate Studies in Orthodontics - Unicsul - São Paulo; 2 PhD in Sciences - UNIFESP, Professor - Graduate Program in Orthodontics of the school of Pindamonhangaba; 3 Full Professor of Pediatric Otorhinolaryngology - Department of Otorhinolaryngology and Head and Neck Surgery - UNIFESP-EPM, Head of the ENT-HNS Department - UNIFESP-EPM (Paulista School of Medicine - Federal University of São Paulo). UNIFESP - EPM

**Keywords:** malocclusion, mouth breathing, tonsillectomy

## Abstract

**Abstract:**

Children with hypertrophic tonsils and adenoids may have adverse effects on dental occlusion, which tend to worsen during the growth period. Diagnosis and early treatment is essential.

**Aim:**

Prospective clinical study to compare the cephalometric measurements before and after adenotonsillectomy in mouth breathing patients.

**Material and Method:**

We had 38 patients of both genders, aged between 7 and 11 years in our sample, broken down into: oral group, 18 patients with obstructive hypertrophy of pharyngeal tonsil and/or palate grade 3 or 4; control group, 20 patients with normal breathing. Angular and linear dental measurements were compared between the groups in a 14 months interval. We used the “t” Student and Wilcoxon tests for unpaired samples, at 5% significance, for statistical purposes.

**Results:**

The sagittal position and axial angle of the lower incisors increased significantly in the group with oral breathing, the sagittal position of the upper incisors increased significantly in the oral group, which still had a significant increase in overbite.

**Conclusion:**

Adenotonsillectomy was very effective in improving some dental measurements, with benefits to growing patients preventing malocclusions from becoming difficult to treat or permanent.

## INTRODUCTION

Lymphoid tissue usually develops quickly after birth; it reaches peak size during early childhood and start to regress at around 8 or 10 years of age^1^. In some children, its overgrowth may cause obstruction in the pharyngeal air tract, which may lead to respiratory, sleep, feeding, speech and swallowing disorders^2^. Children with hyperplastic tonsils and adenoids also tend to have mandibular retroposition, lower incisor teeth towards the lingual side and upper incisors turned to the oral side, increase in overjet and overbite reduction^3^. Some authors have reported such effects on dental occlusion^4^,^5^ as consequences of upper airway obstruction and that, despite self-correction being uncommon; there may be significant improvements in the person's dentition after adenotonsillectomy.

Patients who were treated non-surgically maintained or worsened their morphological disorders^5^. Malocclusions tend to get worse upon school age^6^. Recent publications have stressed that children with nasal-respiratory obstructions tend to have vertical facial growth, cross bite, increase in overjet and dentition narrowing^7^. There is consensus among the authors that children with hyperplastic adenoids and palatine tonsils may have impaired facial growth, thus early diagnosis and treatment are paramount.

The goal of the present study was to compare the spontaneous dental effects before and after 14 months of adenotonsillectomy in patients with adenotonsillar hyperplasia.

## MATERIALS AND METHODS

This study has been assessed and approved by the Ethics in Research Committee of the teaching institution where it was executed, under protocol number 0427/04.

**Figure 1:**
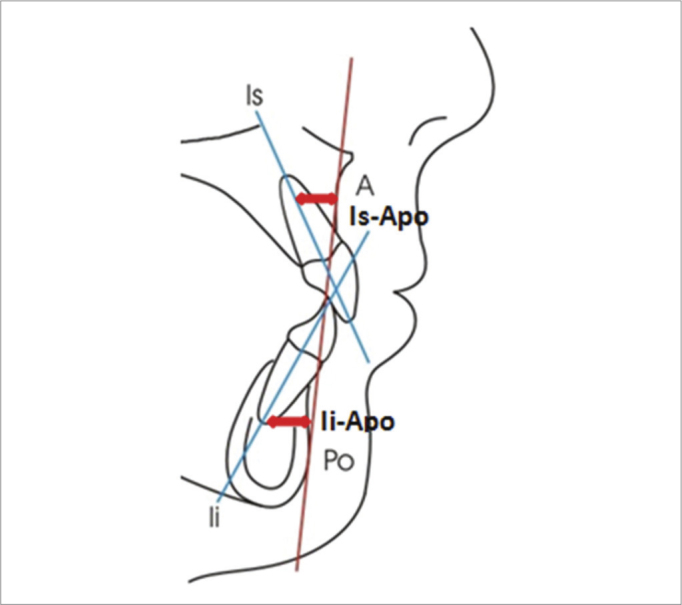
Linear measures which analyzes the upper (Is-Apo) and lower (Ii-Apo) incisor positioning in regards to the Apo reference line.
3 Statistical analysis: for statistical analysis purposes, we employed the Kolmogorov-Smirnov test in order to check for sample symmetry, which did not follow the Gaussian curve. We compared the dental measures within the 14 month interval by means of the t-Student test and the Wilcoxon for unpaired samples, with a 5% significance level. The Student's t-test was used to compare the measures when the normal distribution assumption was confirmed, while the Wilcoxon test was used to compare the measures in which no normal distribution was seen.

1 Sample: made up of 38 patients from both genders, with ages varying between 7 and 11 years, broken down into two groups:
  - Group 1 or Oral Breathing Group: 18 patients with nasal-fibroscopic diagnosis of obstructive hypertrophy of the pharyngeal tonsils (taking up 70% of the choanal space, according to Chami^8^) and/or palatine tonsils hypertrophy grade 3 or 4 (obstruction of 50% to 75% of the air tract in the oropharynx; or more than 75% of air passage obstruction in the oropharynx, according to Brodsky^9^). After the diagnosis, all the patients from this group were submitted to adenotonsillectomy, indicated by an ENT physician.
  - Group 2 or Control Group: 20 patients with nasal breathing, proven by nasal fibroscopic exam, carried out in this institution.
    We took off those patients previously submitted to orthodontic and/or orthopedic treatment from both groups, and also those with maxillomandibular malformations.
2 Dental analysis: the measures were obtained by means of lateral cephalometric radiographies. The Group 1 patients were radiographed before and 14 months after surgery; and group 2 patients were radiographed within a 14 month interval. Figure 1 depicts the measures which were carried out:
  - Axial position of the lower incisor (Ii.Apo): angle formed between the long axis of the inferior incisor and the Apo line, in the axial direction. The Apo line is formed by the A point (point of the largest maxilla concavity) and the Po point (the anterior most point of the chin symphysis).
  - Anteroposterior position of the inferior incisor (Ii-APo): distance in millimeters between the incisal border of the lower incisor and the Apo line, in the sagittal direction.
  - Axial position of the upper incisor (Is.Apo): angle between the upper incisor long axis and the Apo line, in the axial direction.
  - Anteroposterior position of the upper incisor (Is-Apo): distance, in millimeters between the incisal border of the upper incisor tooth and the Apo line, in the sagittal direction.
  - Overjet: distance between the oral face of the upper incisor and the oral face of the lower incisor, in the sagittal direction.
  - Overbite: distance between the incisal border of the upper and lower incisor tooth in the vertical direction.

## RESULTS

**Figure 2:**
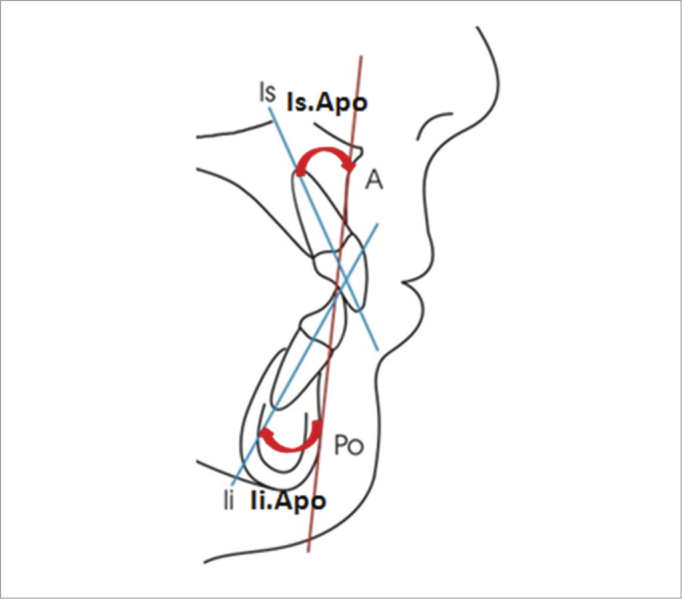
Angular measures which analyze the upper (Is.Apo) and lower (Ii.Apo) incisor tilt in relation to the Apo reference line.

**Figure 3:**
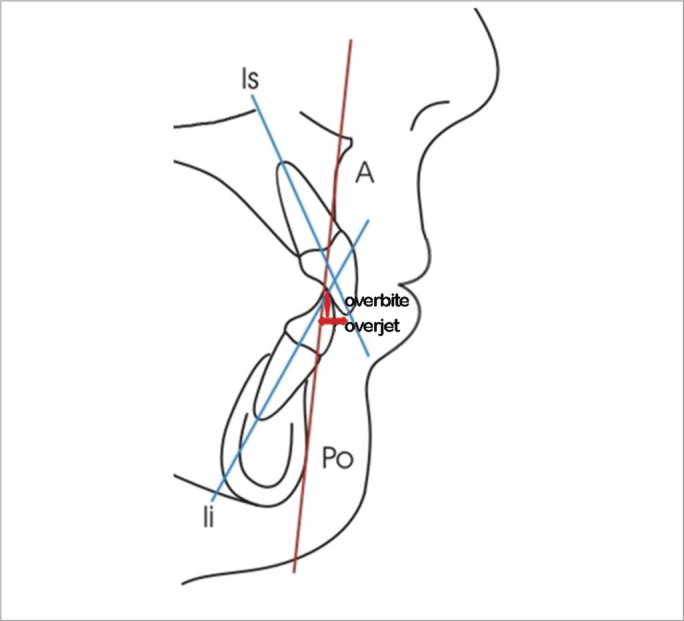
Overbite (vertical measure) and overjet (horizontal measure tg).

Table 1 shows the mean and standard deviation values (according to conventional presentation with the “±” symbol) of the dental variables considered. The measures from the oral group were studied before and after surgical intervention, within 14 months. The nasal group was assessed before and after the same time interval, for control purposes and to compare the groups. Table 2 has the p values found for each comparison, which may be significant for an alpha level of 0.05 or 0.01. Figures 1, 2 and 3 depict the linear and angular variables, overjet and overbite, respectively.

**Table 1:** Cephalometric mean and standard deviation values.

Measures	Oral		Nasal	
	Initial	Final	Initial	Final
Ii-APo (mm)	18.6 ± 4.8	20.9 ± 4.4	23.1 ± 4.8	23.0 ± 5.4
Is.APo (°)	2.7 ± 1.7	3.5 ± 1.9	3.6 ± 2.2	3.2 ± 2.0
Is-APo (mm)	28.8 ± 5.3	29.3 ± 5.5	28.7 ± 6.1	30.8 ± 6.5
Overjet (mm)	6.7 ± 2.7	7.6 ± 2.3	6.8 ± 2.2	6.8 ± 2.3
Overbite (mm)	4.2 ± 2.7	4.3 ± 2.3	3.4 ± 1.5	3.9 ± 1.4
Sobremordida (mm)	-0.7 ± 2.7	1.5 ± 1.8	1.8 ± 2.5	2.0 ± 2.1

**Table 2:** *p* values obtained from the Student's t-test and Wilcoxon intergroup for the initial and final values (Oral *versus* Nasal groups), and Oral and Nasal intergroup (initial *versus* final value).

Measures	Initial	Final	Oral	Nasal
Ii.APo (°)	0.007	0.206	0.039	0.909
Ii-APo (mm)	0.186	0.554	0.007	0.084
Is.APo (°)	0.963	0.098	0.590	0.031
Is-APo (mm)	0.807	0.319	0.050	0.867
Overjet (mm)	0.309	0.522	0.909	0.172
Overbite (mm)	0.004	0.456	0.000	0.466

The first column on Table 2 compares the mean of all the initial values from the oral group, with the mean among all the initial values of the Nasal Group, aiming at checking whether or not the groups were different in the beginning of the study. We noticed that the Ii.APo (^o^) variables and the overbite were significant, whereas the Oral Group had initially less oral tilt and less overbite when compared to the Nasal Group.

The second column on Table 2 compares the mean of all the final values found; and we noticed that there were no differences between the groups, in which the values found for the Oral Group came close to those from the Control Group, after adenotonsillectomy and 14 months had passed before this new measuring.

The third column on Table 2 compared the mean values of all the initial values with the mean values of all the final values only in the Oral Group. We noticed that there were differences between the beginning and the end, before and after adenotonsillectomy, with an increase in the Ii.Apo (^o^), Ii-Apo (mm), Is-Apo (mm) and overbite values - which were statistically significant.

The fourth column of Table 2 compares the mean value from all the initial values with the mean from all the final values of the Nasal Group, with a statistical difference for the Is.Apo (^o^) measure, which increase was significant.

## DISCUSSION

Literature tells us that the assessment of dental measures between oral and nasal breathers involving the position of the incisor teeth has controversial results. Linder-Aronson et al.^10^ noticed in oral breathers the presence of a greater oral tilt of the upper incisors in relation to the NS line (Nasium-Sela). Notwithstanding, authors such as Tarvonen & Koski^11^ and Faria et al.^12^ did not find differences in the positioning of the upper incisors. When we compared the angular and linear positioning of the incisor teeth of our series, we noticed that in the beginning of the study the oral group had a sagittal tilt, which was significantly lower than that of the Nasal Group, and after adenotonsillectomy we did not find differences in the positioning of the incisor teeth in the Oral group of patients who were surgically treated (Table 2, columns 1 and 2). Both angular and linear measures of the Oral Group were farther from the clinical normality in the beginning of the study, and it may indicate that the normalizing of the respiratory pattern, brought about by adenotonsillectomy, has possibly favored the posture and functioning of the orofacial muscles, with a consequent morphological balance and improvement in dental positioning.

As far as overbite is concerned, we noticed that the initial measures of the Oral Group were significantly lower (negative) in relation to those from the Nasal Group, which indicates that this group has patients with anterior open bite. This result is in agreement with studies from other authors, such as Linden-Aronson^13^, Bresolin et al.^14^, Cheng et al.^15^, and Trotman et al.^16^, who equally observed dental infraocclusion in oral breathers. The final mean values do not show differences between the two groups studied, which indicates overbite correction after surgery. According to Jefferson^17^, when oral breathing is treated early on, its deleterious effects on the dental-facial skeleton may be reduced or even prevented. Thus, should the patients in the sample not have been treated early, it is very likely that their open bite would maintain or it would get worse as they grew.

As we compare the dental effects which happened to the oral group after adenotonsillectomy (intragroup), we noticed significant differences in the positioning and sagittal angle of the incisors, and also on the overbite, with all the values tending towards clinical normality. The Nasal Group showed a difference only in the sagittal tilt of the upper incisor, very likely the result of the person's own growth during the time interval of the study; such data points to the need for an orthodontic follow up for all the patients who are in the facial growth stage.

This study showed that adenotonsillectomy brought about benefits in relation to dental occlusion, as it favors the morpho-functional development of the face. Nonetheless, it is important that the orthodontic follow up happens all the way to the final phase of the facial growth, because some cases may need orthodontic treatment associated. The contribution of otorhinolaryngologists, orthodontists and speech and hearing therapists is strongly recommended for the treatment of oral breathing^18^.

## CONCLUSIONS

After adenotonsillectomy, we could notice the following spontaneous dental effects:

1 The axial tilt and the sagittal position of the lower incisor teeth increased significantly in the oral breathing group, tending towards clinical normality.
2 The sagittal positioning of the upper incisor teeth increased significantly in the group submitted to adenotonsillectomy, tending towards clinical normality.
3 The oral group had a significant increase in overbite, which means improvements in the trend towards anterior open bite the patient had before the adenotonsillectomy.
4 Adenotonsillectomy proved efficient to improve some dental measures, which benefits patients who are in their growth phase; preventing dental malocclusions from becoming difficult to treat or even becoming permanent.
